# The Incidence of Alpha-Thalassemia in Iraqi Turks

**DOI:** 10.5505/tjh.2012.48751

**Published:** 2012-03-05

**Authors:** Duran Canatan

**Affiliations:** 1 Hemoglobinopathy Diagnosis Center of Mediterrenaean Blood Diseases Foundation, Antalya, Turkey

## TO THE EDITOR

I read the article on the “The incidence of alpha-thalassemiain Iraqi Turks”. Esmale et al showed that 8 ofthe 83 participants were diagnosed with alpha-thalassemiaan incidence rate of 9.6% and particularly 3.7 kb deletionin Iraqi Turkmens [1].

Although the incidence studies of alpha thalassemia inTurkey are rare, more frequently hemoglobin H diseasehas been observed in south of Turkey, The molecular basisof Hb H disease was studied and mutations - α3.7, -α4.2,-MED-I and - α 20.5 were found to be responsible for thedisease [2,3].

The frequency of alpha-thalassemia was 3.6% amongTurkish newborns in a study that employed globin genemapping analysis of DNA [4]. In our study, in 13 out of205 cord blood samples alpha-thalassemia was found6.3%. There was mistake at incidence so hat we improvedwith erratum [5]. The incidence of β-thalassemia traitwas very high level, the incidence of α-thalassemia traitwas also found high level (6.3%) in Antalya district.

We are observing a lot of uncertain couples with alphathalassemia in premarital screening tests in hemoglobinopathydiagnosis center of in Antalya, it needs moleculardiagnostic test to all of them.

As conclusion, the incidence of alpha-thalassemia wasmuch higher in the Iraqi Turks in the present study thanthat reported in studies from Turkey. It needs more moleculardiagnostic studies in especially adults in Turkey.

## CONFLICT OF INTEREST STATEMENT

The authors of this paper have no conflicts of interest,including specific financial interests, relationships, and/or affiliations relevant to the subject matter or materialsincluded.

## References

[1] Esmael A, Öztürk A, Akar N (2011). The incidence of alphathalassemia in Iraqi Turks. Turk J Haematol.

[2] Oner C, Gürgey A, Oner R, Balkan H, Gümrük F, Baysal E, Altay C (1997). The molecular basis of Hb H disease in Turkey. Hemoglobin.

[3] Taştan AÖ, Canatan D, Başak AN (1999). A search for the α-thal-2 determinants -α3.7 and -α4.2 in the newborns from Antalya district of Turkey: Cord blood study using the PCR method. Balkan Journal of Medical Genetics.

[4] Fei YJ, Kutlar F, Harris HF 2nd, Wilson MM, Milana A, Sciacca P, Schiliro G, Masala B, Manca L, Altay C (1989). A search for anomalies in the zeta, alpha, beta and gamma globin gene arrangements in normal black, Italian, Turkish, and Spanish newborns. Hemoglobin.

[5] Canatan D, Oğuz N, Güvendik İ, Yıldırım S (2002). The Incidence of Alpha-Thalassemia in Antalya-Turkey. Turk J Haematol.

